# Interface dynamics in planar neural field models

**DOI:** 10.1186/2190-8567-2-9

**Published:** 2012-05-02

**Authors:** Stephen Coombes, Helmut Schmidt, Ingo Bojak

**Affiliations:** 1 School of Mathematical Sciences, University of Nottingham, Nottingham, NG7 2RD, UK; 2 School of Psychology (CN-CR), University of Birmingham, Edgbaston, Birmingham, B15 2TT, UK; 3 Centre for Neuroscience, Donders Institute for Brain, Cognition and Behaviour, Nijmegen, 6500 HB, The Netherlands

## Abstract

Neural field models describe the coarse-grained activity of populations of interacting neurons. Because of the laminar structure of real cortical tissue they are often studied in two spatial dimensions, where they are well known to generate rich patterns of spatiotemporal activity. Such patterns have been interpreted in a variety of contexts ranging from the understanding of visual hallucinations to the generation of electroencephalographic signals. Typical patterns include localized solutions in the form of traveling spots, as well as intricate labyrinthine structures. These patterns are naturally defined by the interface between low and high states of neural activity. Here we derive the equations of motion for such interfaces and show, for a Heaviside firing rate, that the normal velocity of an interface is given in terms of a non-local Biot-Savart type interaction over the boundaries of the high activity regions. This exact, but dimensionally reduced, system of equations is solved numerically and shown to be in excellent agreement with the full nonlinear integral equation defining the neural field. We develop a linear stability analysis for the interface dynamics that allows us to understand the mechanisms of pattern formation that arise from instabilities of spots, rings, stripes and fronts. We further show how to analyze neural field models with linear adaptation currents, and determine the conditions for the dynamic instability of spots that can give rise to breathers and traveling waves.

## 1 Introduction

The functional organization of cortex appears to be roughly columnar, with the laminar sub-structure of each column organizing its micro-circuitry. These columns tessellate the two-dimensional cortical sheet with high density, e.g., 2,000 cm^2^ of human cortex contain 10^5^ to 10^6^ macrocolumns, comprising about 10^5^ neurons each. Neural field models describe the mean activity of such columns by approximating the cortical sheet as a continuous excitable medium. They can generate rich patterns of emergent spatiotemporal activity and have been used to understand visual hallucinations, mechanisms for short term working memory, motion perception, the generation of electroencephalographic signals and many other neural phenomena. We refer the reader to [1,2] for recent discussions of neural field models and their uses, and in particular to the work of Bressloff and colleagues [3-5] and Owen et al. [6] for results on planar systems. A minimal two-dimensional neural field model can be written as an integro-differential equation of the form

(1) $$u_{t}(x,t)=-u(x,t)+\int_{R^{2}}w\left(x-x^{\prime }\right)H\left(u\left(x^{\prime },t\right)-h\right)\;dx^{\prime },$$

where $x\in R^{2}$ and $t\in R^{+}$. Here the variable *u* represents synaptic activity and the kernel *w* represents anatomical connectivity. The nonlinear function *H* represents the firing rate of the tissue and will be taken to be a Heaviside so that the parameter *h* is interpreted as a firing threshold. For the case of a symmetric synaptic kernel w(x)=w(|x|), the model also has a Liapunov function [6,7] given by

(2) $$\begin{array}{rl} E_{Liap.}[u] & =-\frac{1}{2}\int dx\int dx^{\prime }w\left(|x-x^{\prime }|\right)H\left(u(x,t)-h\right)H\left(u\left(x^{\prime },t\right)-h\right)\\ & \;+h\int dxH\left(u(x,t)-h\right), \end{array}$$

which can be useful in determining the stability of equilibrium solutions.

**Fig. 1:**
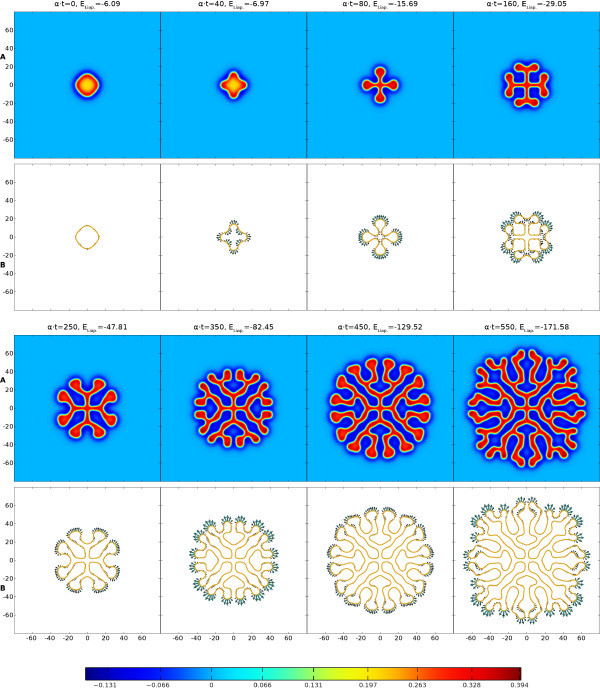
Labyrinthine structure emerging from (1) and (20) with parameters β=0.5, γ=4 and Heaviside threshold h=0.115. The initial spot of radius R=12 has a mode four instability, cf. Figure 4. This is primed by perturbing *R* with 0.5cos(4θ). Rows **A** show *u* and the colorbar below indicates its values. Rows **B** illustrate the evolution of the interface (u=h, golden outline) due to the normal velocity of the boundary (*green arrows*, to scale but enlarged by a factor 50). The Liapunov function $E_{Liap.}$ of (2) is noted at all eight time steps. See also the video in Additional file 1.

Neural field models support traveling waves that underlie EEG signals; but also spots of localized high firing activity, which have been linked to models of working memory. These spots can become unstable and can pattern cortex with intricate structures. In Figure 1A we show results of a direct numerical simulation with a classic Mexican-hat choice for *w*. For further details see the discussion around Equation (20) and Section A.1 in the Appendix (for the numerical scheme). Here Equation (1) describes a single population model with short-range excitation and long-range inhibition. This minimal example nicely illustrates the ability of neural field models to generate intricate spreading labyrinthine patterns. We do not expect to find labyrinthine patterns as such in real brain activity. However, they provide a convenient (and visually striking) proxy for the generation of complex patterns of activity, that emerge spontaneously and/or can be evoked, for example in visual cortex [8]. Labyrinthine patterns are also seen when the Heaviside firing rate function is replaced by a steep sigmoid, as will be discussed later. Visual inspection suggests that much of the behavior of such patterns can be described simply by tracking the boundary between high and low states of activity. Indeed this appears to resonate with neuroscientific practice, where changes of brain activity are often of greater interest than the current brain state *per se*[9]. Hence it is of interest whether the dynamics of (1) can be replaced by a lower dimensional description that evolves the boundary between high and low states of activity. This programme has already been developed by Amari in his seminal article on one-dimensional models [10], where this interface reduces naturally to a point (or a set of points). However, in two spatial dimensions the interface is more naturally a closed curve (or a set of closed curves). 

The main topic of this article is the development of an equivalent interface description for neural field models of the type exemplified by (1). We show that activity patterns can be described by dynamical equations of reduced dimension, and that these depend only on the shape of the interface (requiring no knowledge of activity away from the interface). Not only is this description amenable to fast numerical simulation strategies, it allows for the construction of localized states and an analysis of their linear stability. Given the computational overheads in simulating the full neural field model this enhances our ability to study pattern formation and suggests more generally that modeling the interfaces of patterns, rather than the patterns themselves, may lead to novel, efficient descriptions of brain activity. Indeed the use of interface dynamics to analyze patterns that arise in partial differential equation models of chemical and physical systems has a strong history [11], and it is natural to translate some of the ideas and technologies from these studies to non-local neural field models. The work by Goldstein [12,13] and Muratov [14] on pattern formation in two-dimensional excitable reaction-diffusion systems is especially relevant in this context, as both authors have developed effective descriptions of interface dynamics in terms of non-local interactions. See also the book by Desai and Kapral [15] for a recent overview. 

It is worth pointing out that whether computing interface dynamics can compete with other numerical schemes will depend on the problem at hand. In general, boundaries that remain relatively short and do not pinch guarantee a speed advantage. In practice, we expect this approach to be especially relevant for (semi-) analytical work aiming at qualitative understanding, as illustrated by some of the examples presented in this article.

In Section 2 we present some of the key ideas behind an interface dynamics in the setting of a one-dimensional neural field model. This is particularly useful for introducing the definition of normal velocity from a level-set condition, as well as establishing what it means for an interface to be linearly stable. The extension of these ideas to two-dimensional systems is presented in Section 3. By writing the synaptic connectivity in terms of a linear combination of Bessel functions, we show that dynamics for the interface can be constructed in terms of line-integrals along the interface, and that the normal velocity of the interface is driven by Biot-Savart-style interactions. Thus we obtain a reduced description for the evolution of a pattern boundary solely in terms of quantities on the boundary itself. Numerical simulations of the interface dynamics are shown to be in direct correspondence with those of the full neural field model. The notion of linear stability of stationary solutions in the interface framework is fleshed out in a series of examples (for spots, rings, stripes and fronts) in Sections 4 and 5, and allows us to understand some of the mechanisms for pattern formation. In Section 6 we add linear adaptation to (1) and extend our analysis to cover this important neural phenomenon. This can introduce dynamic instabilities of stationary structures, and we calculate where breathing and drift instabilities for localized spots occur. Moreover, we use a perturbation argument to determine the shape of traveling spots that emerge beyond a drift instability and show that spots contract in the direction of propagation and widen in the orthogonal direction. Finally, in Section 7 we discuss extensions of the work in this article.

## 2 A one-dimensional primer

Before we develop the machinery for describing the evolution of interfaces in two-dimensional neural field models, it is informative to begin with a discussion in one dimension. In this case a minimal model can be written in the form

(3) $$u_{t}=-u+\psi ,\;\psi (x,t)=\int_{R}w(x-y)H\left(u(y,t)-h\right)\;dy,$$

where u=u(x,t) and x∈R, $t\in R^{+}$. For a symmetric choice of synaptic kernel w(x)=w(|x|), which decays exponentially, the one-dimensional model (3) is known to support a traveling front solution [16,17] that connects a high activity state to a low activity state. In this case it is natural to define a pattern boundary as the interface between these two states. Thus we can define a moving interface (level set) according to

(4) $$u\left(x_{0}(t),t\right)=\text{const}.$$

Here we are assuming that there is only one point on the interface, though in principle we could consider a set of points. The function $x_{0}=x_{0}(t)$ gives the evolution of the interface. Since the high and low activity states in the neural field model are naturally distinguished by determining whether *u* is above or below the firing threshold, we shall take the constant on the right hand side of (4) to be *h* (though other choices are also possible). Differentiation of (4) gives an exact expression for the velocity of the interface in the form

(5) $${\dot{x}}_{0}=-\frac{u_{t}}{u_{x}}{|}_{x=x_{0}(t)}.$$

We can now describe the properties of a front solution solely in terms of the behavior at the front edge which separates high activity from low. To see this, let us assume that the front is such that u(x,t)>h for $x<x_{0}(t)$ and u(x,t)≤h for $x\geq x_{0}(t)$. Then (3) reduces to

(6) $$u_{t}(x,t)=-u(x,t)+\int_{x-x_{0}(t)}^{\infty }w(y)\;dy.$$

Introducing $z=u_{x}$ and differentiating (6) with respect to *x* gives

(7) $$z_{t}(x,t)=-z(x,t)-w\left(x-x_{0}(t)\right).$$

Integrating (7) from −∞ to *t* (and dropping transients) yields

(8) $$z(x,t)=-e^{-t}\int_{-\infty }^{t}e^{s}w\left(x-x_{0}(s)\right)\;ds.$$

We may now use the interface dynamics defined by (5) to study the speed c>0 of a front, defined by ${\dot{x}}_{0}=c$. In this case $x_{0}(t)=ct$, where without loss of generality we set $x_{0}(0)=0$, and from (6) and (8) we have that

(9) $$u_{t}{|}_{x=x_{0}(t)}=-h+\tilde{w}(0),\;u_{x}{|}_{x=x_{0}(t)}=-\tilde{w}(1/c)/c,$$

where

(10) $$\tilde{w}(\lambda )=\int_{0}^{\infty }e^{-\lambda s}w(s)\;ds.$$

Hence from (5) the speed of the front is given implicitly by the equation

(11) $$h=\tilde{w}(0)-\tilde{w}(1/c).$$

To determine stability of the traveling wave we consider a perturbation of the interface and an associated perturbation of *u*. Introducing the notation $\hat{\cdot }$ to denote perturbed quantities, to a first approximation we will set ${\hat{u}}_{x}{|}_{x={\hat{x}}_{0}(t)}=u_{x}{|}_{x=ct}$, and write ${\hat{x}}_{0}(t)=ct+\delta x_{0}(t)$. The perturbation in *u* can be related to the perturbation in the interface by noting that both the perturbed and unperturbed boundaries are defined by the level set condition, so that $u(x_{0},t)=h=\hat{u}({\hat{x}}_{0},t)$. Introducing $\delta u(t)=u{|}_{x=ct}-\hat{u}{|}_{x={\hat{x}}_{0}(t)}$, we thus have the condition that δu(t)=0 for all *t*. Integrating (6) and dropping transients gives

(12) $$u(x,t)=e^{-t}\int_{-\infty }^{t}dse^{s}\int_{x-x_{0}(s)}^{\infty }dyw(y),$$

and $\hat{u}$ is obtained from (12) by simply replacing $x_{0}$ by ${\hat{x}}_{0}$. Using the above we find that *δu* is given (to first order in $\delta x_{0}$) by

(13) $$\delta u(t)=\frac{1}{c}\int_{0}^{\infty }dse^{-s/c}w(s)\left[\delta x_{0}(t)-\delta x_{0}(t-s/c)\right]=0.$$

This has solutions of the form $\delta x_{0}(t)=e^{\lambda t}$, where *λ* is defined by E(λ)=0, with

(14) $$E(\lambda )=1-\frac{\tilde{w}((1+\lambda )/c)}{\tilde{w}(1/c)}.$$

A front is stable if Reλ<0.

As an example consider the choice w(x)=exp(−|x|/σ)/(2σ), for which $\tilde{w}(\lambda )={\left(\lambda +1/\sigma \right)}^{-1}/(2\sigma )$. In this case the speed of the wave is given from (11) as

(15) $$c=\sigma \frac{1-2h}{2h},$$

and

(16) $$E(\lambda )=\frac{\lambda }{1+c/\sigma +\lambda }.$$

The equation E(λ)=0 only has the solution λ=0. We also have that $E^{\prime }(\lambda )>0$, showing that λ=0 is a simple eigenvalue. Hence, the traveling wave front for this example is neutrally stable.

Given this preliminary exposition of interface dynamics we are now ready to describe the extension to two dimensions and to address the additional challenges that working in the plane gives rise to.

## 3 Interface dynamics in two dimensions

As in the one-dimensional case we will define pattern boundaries as the interface between low and high states of neural activity. To be more precise we introduce the notation B(t) to denote the (compact) area of activity where u≥h. The boundary, or interface, ∂B(t) is defined by the threshold crossing condition u(x,t)=h. In this case the model defined by (1) takes the form

(17) $$u_{t}(x,t)=-u(x,t)+\psi (x,t),\;\psi (x,t)=\int_{B(t)}w\left(|x-x^{\prime }|\right)\;dx^{\prime },$$

and the Liapunov function can be written simply as

(18) $$E_{Liap.}[u]=-\frac{1}{2}\int_{B(t)}dx\int_{B(t)}dx^{\prime }w\left(|x-x^{\prime }|\right)+h\int_{B(t)}dx.$$

**Fig. 2:**
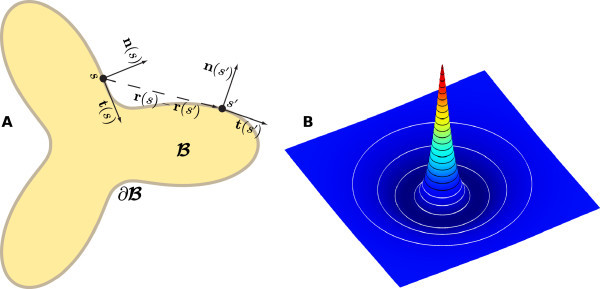
Interface parametrization and Mexican-hat shape for synaptic kernel. **A**: Compact area B and boundary ∂B. Two points on the boundary **r**, parametrized by *s* and $s^{\prime }$, are shown with their normal **n** and tangent **t** vectors. **B**: Mexican hat (20) with parameters β=0.3 and γ=3. White contours indicate values <0, black ones ≥0, for a step size of 0.005.

Note that B(t) does not have to be simply connected and can describe a union of many disjoint active regions. However, for clarity of exposition we shall focus on describing the evolution of an interface that is a single closed curve, as depicted in Figure 2A. The extension to multiple closed curves is straight-forward. 

It is well known that the two-dimensional model (17) can support localized states such as spots and rings [18,19] for a Mexican-hat synaptic connectivity. Recent work in [6] has shown how to determine the stability of such solutions to angular perturbations using an Evans function approach [20]. An analogous numerical study for smooth sigmoidal firing rates can be found in [21]. These studies have highlighted, as compared to the one-dimensional model, that an extra spatial dimension can lead to azimuthal instabilities, whereby localized states can deform (or even split) into patterns with a reduced symmetry predicted by the shape of the most unstable eigenmode. Direct numerical simulations beyond such instability points have further shown the emergence of intricate spreading labyrinthine patterns like those in Figure 1, that leave behind a stable patterned state in their wake. It is our intention here to recover the Evans function results for stability, albeit using a purely interface description of dynamics, as well as to determine the nonlinear equations of motion that govern the evolution of labyrinthine (and other) structures. Moreover, by employing a representation of the synaptic connectivity in terms of a linear combination of Bessel functions, we can obtain an exact, though spatially reduced, dynamical system to describe the interface that depends solely on the shape of the interface itself. In the following, we consider kernels of the form [3]

(19) $$w(r)=\sum_{i=1}^{N}A_{i}K_{0}(\alpha_{i}r),\;A_{i}\in R,\alpha_{i}>0,$$

where $K_{0}$ is the zeroth order modified Bessel function of the second kind. In particular we will employ the Mexican-hat shape obtained from

(20) $$w(r)=\frac{2}{3\pi }\left(K_{0}(r)-K_{0}(2r)-\frac{1}{\gamma }\left(K_{0}(\beta r)-K_{0}(2\beta r)\right)\right),\;\beta ,\gamma >0,$$

which is shown for β=0.3 and γ=3 in Figure 2B.

In an identical fashion to the way we derived an interface dynamics in one dimension in Section 2, we differentiate u(x,t)=h along the contour ∂B(t) to obtain

(21) $$\nabla_{x}u\cdot \frac{dr}{dt}+\frac{\partial u}{\partial t}=0,$$

where **r** is a point on the domain boundary ∂B and $u_{t}$ and $\nabla_{x}u$ are evaluated on the boundary. Introducing the normal vector along the contour ∂B as $n=-\nabla_{x}u/|\nabla_{x}u|$ allows us to obtain the normal velocity along the contour:

(22) $$n\cdot \frac{dr}{dt}=\frac{u_{t}}{\left|z\right|},$$

where $z\equiv \nabla_{x}u(x,t){|}_{x=r}$. Using (17) we see that *u* and *z* satisfy

(23) $$u_{t}=-h+\int_{B}dx^{\prime }w\left(|r-x^{\prime }|\right),$$

(24) $$z_{t}=-z+\nabla_{x}\int_{B}dx^{\prime }w\left(|x-x^{\prime }|\right){|}_{x=r}.$$

From the form of (22), (23), and (24), we see that the evolution of the interface does not require any knowledge of the neural field away from the contour, and rather just depends on the shape of the sets where the field is above threshold. We now exploit the choice of $K_{0}$ as basis function for constructing the synaptic kernel to show how the double integrals in (23) and (24) can be reduced to line integrals. This yields an elegant description of the interface dynamics that emphasizes how the geometry of ∂B drives the evolution of spatiotemporal patterns. The key step in this reformulation is the use of Green’s identity. For a two-dimensional vector field **F** this identity is the two-dimensional version of the divergence theorem, which we write symbolically as $\int_{B}\nabla \cdot F=\oint_{\partial B}F\cdot n$. Using this first identity we may generate a second for a scalar field Ψ as $\int_{B}\nabla \Psi =\oint_{\partial B}n\Psi$.

To evaluate the right hand side of (23) and (24) it is enough to calculate $\int_{B}dx^{\prime }K_{0}(\alpha |x-x^{\prime }|)$ and its gradient. In fact, this latter term can easily be rewritten as a line integral, using the second Green’s identity, for any choice of synaptic kernel

(25) $$\begin{array}{rl} \int_{B}dx^{\prime }\nabla_{x}w\left(\left|x-x^{\prime }\right|\right) & =-\int_{B}dx^{\prime }\nabla_{x^{\prime }}w\left(\left|x-x^{\prime }\right|\right)\\ & =-\oint_{\partial B}dsn(s)w\left(\left|x-x^{\prime }(s)\right|\right). \end{array}$$

Using the fact that $K_{0}(\alpha x)$ satisfies the identity $K_{0}(\alpha x)=\alpha^{-2}\nabla^{2}K_{0}(\alpha x)+2\pi \delta (\alpha x)$, as well as $\nabla_{x}w(|x|)=w^{\prime }(|x|)x/|x|$ and $K_{0}^{\prime }=-K_{1}$, an application of Green’s first identity shows that

(26) $$\begin{array}{rcl} & & \int_{B}dx^{\prime }K_{0}\left(\alpha \left|x-x^{\prime }\right|\right)\\ & & \;=\frac{1}{\alpha^{2}}\int_{B}dx^{\prime }\nabla_{x}^{2}K_{0}\left(\alpha \left|x-x^{\prime }\right|\right)+2\pi \int_{B}dx^{\prime }\delta \left(\alpha \left|x-x^{\prime }\right|\right)\\ & & \;=-\frac{1}{\alpha }\oint_{\partial B}dsn(s)\cdot \frac{x-r(s)}{\left|x-r(s)\right|}K_{1}\left(\alpha \left|x-r(s)\right|\right)+C\frac{2\pi }{\alpha^{2}}. \end{array}$$

Here C=1 if **x** is within B and C=0 if **x** is outside B. If **x** is on the boundary of B then C=1/2. Hence, for points on the boundary parametrized by *s* one finds

(27) $$u_{t}(s)=-h+\sum_{i=1}^{N}A_{i}\left\{\oint_{\partial B}ds^{\prime }n\left(s^{\prime }\right)\cdot R_{i}\left(s,s^{\prime }\right)+\frac{\pi }{\alpha_{i}^{2}}\right\},$$

(28) $$z_{t}(s)=-z(s)-\oint_{\partial B}ds^{\prime }n\left(s^{\prime }\right)w\left(\left|r(s)-r\left(s^{\prime }\right)\right|\right),$$

where

(29) $$R_{i}\left(s,s^{\prime }\right)=-\frac{1}{\alpha_{i}}\frac{r(s)-r(s^{\prime })}{\left|r(s)-r(s^{\prime })\right|}K_{1}\left(\alpha_{i}\left|r(s)-r\left(s^{\prime }\right)\right|\right).$$

Note that the choice of $K_{0}$ as a basis for *w* is merely a convenience to allow explicit calculations. As long as we can write the connectivity function *w* as the divergence of a vector field then we can exploit Green’s first identity to turn the right hand side of (23) into a line integral.

From the Biot-Savart form of (29) we see that for every part *i* of the synaptic kernel there is an effective repulsion between two arc length positions with anti-parallel tangent vectors, although the combined effect when including all *N* terms will depend on the choice of the amplitudes $A_{i}$. Now with (22), (27), and (28) the normal velocity on the interface can be written solely in terms of certain line-integrals around the interface. From a computational perspective this leads to a substantial advantage in that one no longer needs to solve the full non-local neural field model (17) across the entire plane, and can instead simply evolve the interface in time by discretizing the boundary and translating the points with the normal velocity from (22) in the direction of **n**. One possible practical disadvantage of this is the need to monitor for possible self-intersections of the evolving boundary, *splitting*, where a connected region pinches off into two or more disconnected regions, or indeed the creation of new boundaries where none existed before. However, numerical schemes for coping with similar situations in fluid models are well developed in the literature and it is natural to turn to these for more refined numerical schemes and ones that can automate the process of contour surgery [22,23]. In Figure 1B we illustrate the simple numerical implementation of the interface dynamics described in Section A.2 in the Appendix, showing the effectiveness of the dimensionally reduced system at capturing the spatiotemporal pattern formation of the full model shown in Figure 1A.

**Fig. 3:**
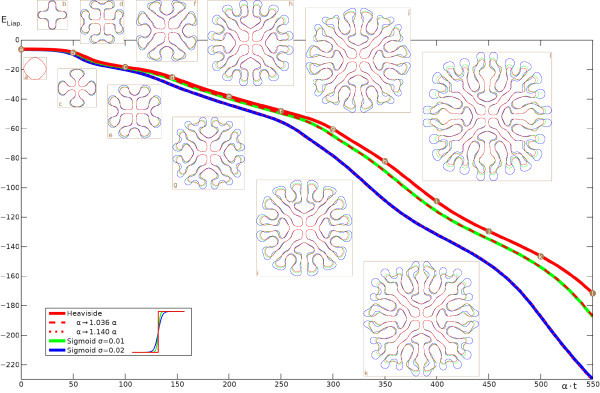
Time dependence of the u=h interface and Liapunov function. *Red curves* are for the Heaviside model of Figure 1, *green* and *blue* ones use a sigmoid instead. *Circular labels* indicate the times of the snapshots. *Dashed* and *dotted curves* scale the Heaviside one by adjusting *α*. See also the video in Additional file 2.

Furthermore, in our calculations we have found that the key assumption of a Heaviside firing rate H(u−h) can be relaxed to a degree without fundamentally changing the results. This is illustrated in Figure 3, where we show the evolution in time of the u=h interface and the corresponding Liapunov function. The evolution with a Heaviside firing rate H(u−h) is shown in red, and compared with simulations of the full neural field model using more biologically realistic sigmoids 1/{1+exp[−(u−h)/σ]}, with σ=0.01 in green and σ=0.02 in blue. Here *σ* reflects the expected width of the distribution of firing thresholds around a mean *h* in the neural population, with the Heaviside case corresponding to σ=0. Figure 3 demonstrates that for these steep sigmoids very similar labyrinthine shapes arise, and closer inspection reveals that the main differences occur at the rapidly developing rim of the structure, whereas the settled interior is nearly identical. Thus a simple adjustment of the time constant *α* will in this case provide a near perfect match of the emerging structures. In Figure 3 we demonstrate this with the dashed and dotted red lines, which represent the Heaviside Liapunov function computed over longer time scales (up to α⋅t=569.9 and 626.8, respectively) and then scaled down to α⋅t≤550 by adjusting *α*. A very close match to the sigmoidal Liapunov curves (green and blue lines) is then obtained. However, for broader sigmoids we find labyrinths still resembling the Heaviside one, but with more obvious spatial changes. The video in Additional file 3 shows the σ=0.03 case as an example. It would seem that mild deviations in the shape of the firing rate from Heaviside (to a steep sigmoidal form) are reflected more in temporal speed than in spatial shape changes. 

The Liapunov function can also be written in terms of line integrals: $E_{Liap.}=1/2\sum_{i=1}^{N}A_{i}F_{i}+h\Gamma$, with

(30) $$F_{i}=\frac{1}{\alpha_{i}^{2}}\oint_{\partial B}ds\oint_{\partial B}ds^{\prime }t(s)\cdot t\left(s^{\prime }\right)K_{0}\left(\alpha_{i}\left|r(s)-r\left(s^{\prime }\right)\right|\right)-\frac{2\pi }{\alpha_{i}^{2}}\Gamma ,$$

where $\Gamma =\int_{B}dx$ is the area of the domain above threshold and t(s)=dr(s)/ds is the tangent vector, which can also be constructed from **n** by an anti-clockwise rotation of π/2 so that

(31) $$n=\left[\begin{array}{cc} 0 & 1\\ -1 & 0 \end{array}\right]t.$$

To obtain (30) we have used the fact that

(32) $$\begin{array}{rl} & \int_{B}dx\int_{B}dx^{\prime }K_{0}\left(\alpha \left|x-x^{\prime }\right|\right)-\frac{2\pi }{\alpha^{2}}\Gamma \\ & \;=-\frac{1}{\alpha^{2}}\int_{B}dx\nabla_{x}\cdot \int_{B}dx^{\prime }\nabla_{x^{\prime }}K_{0}\left(\alpha \left|x-x^{\prime }\right|\right)\\ & \;=-\frac{1}{\alpha^{2}}\int_{B}dx\nabla_{x}\cdot \oint_{\partial B}ds^{\prime }n\left(s^{\prime }\right)K_{0}\left(\alpha \left|x-r\left(s^{\prime }\right)\right|\right)\\ & \;=-\frac{1}{\alpha^{2}}\oint_{\partial B}ds\oint_{\partial B}ds^{\prime }n(s)\cdot n\left(s^{\prime }\right)K_{0}\left(\alpha \left|r(s)-r\left(s^{\prime }\right)\right|\right), \end{array}$$

and the observation that $n(s)\cdot n(s^{\prime })=t(s)\cdot t(s^{\prime })$.

As well as providing a computationally useful framework for studying pattern formation, the interface dynamics including its Liapunov function is also amenable to a direct linear stability analysis. This is especially useful for understanding how the instability of localized stationary states can seed interesting structures, like the labyrinths of Figures 1 and 3. Stationarity of a solution means that the normal velocity is zero all along the boundary of the active area. This is equivalent to demanding $u_{t}=0$ on the boundary. In this case (22) reduces to

(33) $$h=\int_{B_{0}}dx^{\prime }w\left(\left|r-x^{\prime }\right|\right)=\sum_{i=1}^{N}A_{i}\left\{\oint_{\partial B_{0}}ds^{\prime }n\left(s^{\prime }\right)\cdot R_{i}\left(s,s^{\prime }\right)+\frac{\pi }{\alpha_{i}^{2}}\right\},$$

where **r** is on the boundary parametrized by *s*. We use the notation $B_{0}$ to denote a stationary active region. Given the stationary interface, we can also calculate the stationary field *u* everywhere (away from the interface) using (17) as

(34) $$u(x)=\int_{B_{0}}dx^{\prime }w\left(\left|x-x^{\prime }\right|\right),$$

which can also be evaluated as a line integral. In order to analyze the stability of stationary solutions in the original neural field formalism defined by (1) one would perturb the field variable *u* and linearize to derive an eigenvalue equation or Evans function [20]. Here we determine stability using the interface dynamics, generalizing the approach described in Section 2.

Using the notation $\hat{\cdot }$ again to denote perturbed quantities, we consider small perturbations to the contour shape and denote the new interface by $\hat{\partial B}$. The relationship between the perturbed interface and the perturbed field is, as in one dimension, determined by the condition δu(t)=0, where

(35) $$\delta u(t)=\hat{u}{|}_{x\in \hat{\partial B}}-u{|}_{x\in \partial B_{0}}.$$

The dynamics for $\hat{u}$ is given by (23) with B replaced by $\hat{B}$. The perturbation affects the normal vector n(s) as well as the displacement vector $r(s)-r(s^{\prime })$ that occurs in (27). Thus to evaluate (35) it is necessary to linearize $K_{1}$ about the unperturbed contour. In the case of interfaces without curvature the linear contribution to $K_{1}$ is zero. In contrast for curved interfaces an addition theorem for Bessel functions shows that there is a non-zero contribution. To clarify this statement and show how the above machinery is used in practice, we now give some explicit examples of localized solutions and their stability.

## 4 Localized states: spots

 We consider spots to be circular stationary solutions. They are the equivalent of the bumps known in one spatial dimension [10]. For radially symmetric kernels we expect stationary circular solutions. Yet in two spatial dimensions they can undergo *azimuthal* instabilities, as already found in [6]. In order to obtain circular solutions we use the standard parametrization of a circle for the contour and write

(36) $$r(\theta )=R\left[\begin{array}{c} \sin\theta \\ 1-\cos\theta \end{array}\right],\;n(\theta )=\left[\begin{array}{c} \sin\theta \\ -\cos\theta \end{array}\right],\;\theta \in [0,2\pi ).$$

**Fig. 4:**
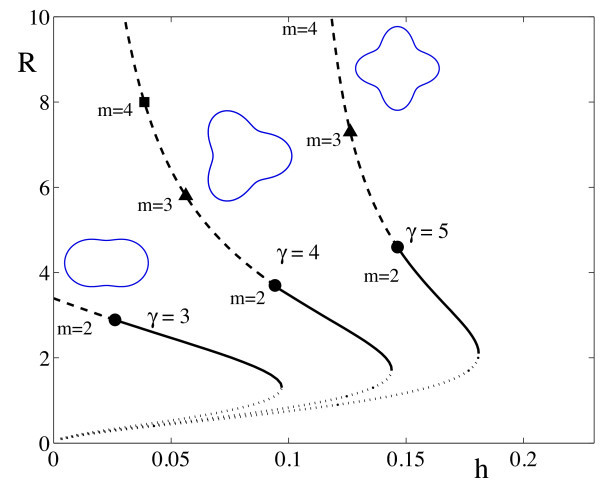
Stationary radial solutions for the Mexican hat kernel (20) with β=0.5 and various values of *γ*. The *dotted branches* are circular solutions unstable to uniform changes of size. *Solid branches* are stable. *Dashed branches* indicate azimuthal instabilities of different modes *m* which deform the circular solution.

Hence the right hand side of (33) can be calculated using

(37) $$\oint_{\partial B}ds^{\prime }n\left(s^{\prime }\right)\cdot R_{i}\left(s,s^{\prime }\right)=\frac{1}{\alpha_{i}}\int_{0}^{2\pi }d\theta \frac{K_{1}[\alpha_{i}R(\theta )]}{R(\theta )}R^{2}(1-\cos\theta ),$$

where $R(\theta )=R\sqrt{2(1-\cos\theta )}$. Using Graf’s formula [24] to perform the integration in (37) we obtain an implicit equation for the spot radius *R* in the form

(38) $$h=2\pi \sum_{i=1}^{N}A_{i}\left\{\frac{1}{\alpha_{i}^{2}}-\frac{R}{\alpha_{i}}K_{1}(\alpha_{i}R)I_{0}(\alpha_{i}R)\right\},$$

where $I_{\nu }(x)$ is the modified Bessel function of the first kind of order *ν*. A plot of the spot radius *R* as a function of threshold *h* is shown in Figure 4. 

To determine the relationship between a perturbed and unperturbed spot we need to examine the condition δu(t)=0. The general solution for *u* (dropping transients) can be written as

(39) $$u(x,t)=e^{-t}\int_{-\infty }^{t}dse^{s}\psi (x,s).$$

For a circular solution of radius *Rψ* is conveniently written as

(40) $$\psi (r)=\int_{0}^{2\pi }\int_{0}^{R}w\left(\left|r-r^{\prime }\right|\right)r^{\prime }dr^{\prime }d\theta ,\;r=(r,\theta ).$$

Here *ψ* may be constructed explicitly (off the boundary), using similar line integral calculations to those for existence (above), and is given by

(41) $$\psi (r)=2\pi R\sum_{i=1}^{N}A_{i}L_{i}(r),$$

where

(42) $$L_{i}(r)=\{\begin{array}{cc} \frac{1}{\alpha_{i}}I_{1}(\alpha_{i}R)K_{0}(\alpha_{i}r), & r\geq R,\\ \frac{1}{\alpha_{i}^{2}R}-\frac{1}{\alpha_{i}}I_{0}(\alpha_{i}r)K_{1}(\alpha_{i}R), & r<R. \end{array}$$

For perturbations in the radius of the form $\hat{R}=R+\delta R(\theta ,t)$ one finds

(43) $$\begin{array}{rcl} \delta u(t) & = & \int_{0}^{\infty }dse^{-s}\int_{0}^{2\pi }d\theta^{\prime }\{\int_{0}^{\hat{R}(\theta^{\prime },t-s)}w\left(\left|r-r^{\prime }\right|\right){|}_{r=(\hat{R}(\theta ,t),\theta )}r^{\prime }dr^{\prime }\\ & & -\int_{0}^{R}w\left(\left|r-r^{\prime }\right|\right){|}_{r=(R,\theta )}r^{\prime }dr^{\prime }\}\\ & = & \int_{0}^{\infty }dse^{-s}\int_{0}^{2\pi }d\theta^{\prime }\left\{\delta R\left(\theta^{\prime }-\theta ,t-s\right)Rw\left(R\left(\theta^{\prime }\right)\right)+\psi^{\prime }(R)\delta R(\theta ,t)\right\}. \end{array}$$

Using the above we see that δu(t)=0 has solutions of the form $\delta R(\theta ,t)=\cos m\theta e^{\lambda_{m}t}$, where

(44) $$\lambda_{m}=-1+W_{m},$$

and

(45) $$W_{m}=\frac{R}{\left|\psi^{\prime }(R)\right|}\int_{0}^{2\pi }d\theta \cos(m\theta )w\left(R(\theta )\right)=\frac{\sum_{i=1}^{N}A_{i}K_{m}(\alpha_{i}R)I_{m}(\alpha_{i}R)}{\sum_{i=1}^{N}A_{i}K_{1}(\alpha_{i}R)I_{1}(\alpha_{i}R)}.$$

Note that since $W_{m}$ is real $\lambda_{m}\in R$. A mode-*m* instability will occur if $\lambda_{m}>0$, which recovers the result in [6] obtained using an Evans function approach. The possibility of such azimuthal instabilities is indicated on the solution branches shown in Figure 4 (and we would expect the emergence of solution branches with $D_{m}$ symmetry from the points marked by *m*). Interestingly we can see from (44) and (45) that the mode with m=1 is neutrally stable. For a perturbation to a circular boundary of the form $\delta R(\theta ,t)=\epsilon_{m}(t)\cos(m\theta )$, $\epsilon_{m}=\epsilon e^{\lambda_{m}t}$ and ϵ≪1, the perturbation of the normal velocity $v_{n}$ is

(46) $$v_{n}=\epsilon_{m}\cos(m\theta )(-1+W_{m}).$$

To calculate the Liapunov function for an unperturbed spot we evaluate (30) using

(47) $$\begin{array}{rl} & \frac{1}{\alpha_{i}^{2}}\int_{0}^{2\pi }d\theta \int_{0}^{2\pi }d\theta^{\prime }R^{2}\cos\left(\theta -\theta^{\prime }\right)K_{0}\left(\alpha_{i}R\left(\theta -\theta^{\prime }\right)\right)\\ & \;=\frac{4\pi^{2}}{\alpha_{i}^{2}}R^{2}K_{1}(\alpha_{i}R)I_{1}(\alpha_{i}R)\equiv G_{i}. \end{array}$$

Hence

(48) $$E_{Liap.}=\frac{1}{2}\sum_{i=1}^{N}A_{i}\left(G_{i}-\frac{2\pi }{\alpha_{i}^{2}}\pi R^{2}\right)+h\pi R^{2}.$$

The zeros of the first derivative of $E_{Liap.}$ with respect to *R* give the stationary circular solutions, including the trivial case R=0, as expected.

## 5 Rings, fronts and stripes

 In this section we show how to treat other simple interface shapes, namely rings, fronts and stripes, and determine their stability. We recover previous results in [6] for rings (obtained with an Evans function method), whilst calculations for the other structures are shown to be straight-forward using the interface dynamics approach. 

### 5.1 Rings

Rings can be considered as the difference of two spots, one with radius $R_{1}$ and the other with radius $R_{2}>R_{1}$. Introducing $\psi (r,R)=2\pi R\sum_{i}A_{i}L_{i}(r,R)$, where $L_{i}(r,R)$ is given by the right hand side of (41), we have that $u(r)=\psi (r,R_{2})-\psi (r,R_{1})$. Enforcing the threshold conditions $u(R_{2})=h=u(R_{1})$ gives a pair of equations that determine $\left(R_{1},R_{2}\right)$. To establish stability the outer contour is perturbed exactly as in the previous section: $R_{2}(\theta )=R_{2}+ae^{\lambda t}\cos m\theta$, for some small amplitude *a*. For the inner contour we similarly write $R_{1}(\theta )=R_{1}+be^{\lambda t}\cos m\theta$. We now generate δu(t) on each of the two boundaries and equate these to zero to generate two equations for the pair of unknown amplitudes (a,b). Demanding that this pair of equations has a non-trivial solution generates an equation for *λ* in the form $E_{m}(\lambda )=0$ where $E_{m}(\lambda )=|(1+\lambda )I_{2}-A_{m}(\lambda )|$ and

(49) $$\begin{array}{rcl} {\left[A_{m}(\lambda )\right]}_{\mu \nu } & = & \frac{R_{\nu }}{\left|u^{\prime }(R_{\nu })\right|}\int_{0}^{2\pi }d\theta \cos(m\theta )w\left(\sqrt{R_{\mu }^{2}+R_{\nu }^{2}-2R_{\mu }R_{\nu }\cos\theta }\right)\\ & = & \frac{R_{\nu }}{\left|u^{\prime }(R_{\nu })\right|}\sum_{i=1}^{N}A_{i}[K_{m}(\alpha_{i}R_{\mu })I_{m}(\alpha_{i}R_{\nu })H(R_{\mu }-R_{\nu })\\ & & +K_{m}(\alpha_{i}R_{\nu })I_{m}(\alpha_{i}R_{\mu })H(R_{\nu }-R_{\mu })], \end{array}$$

for μ,ν=1,2.

**Fig. 5:**
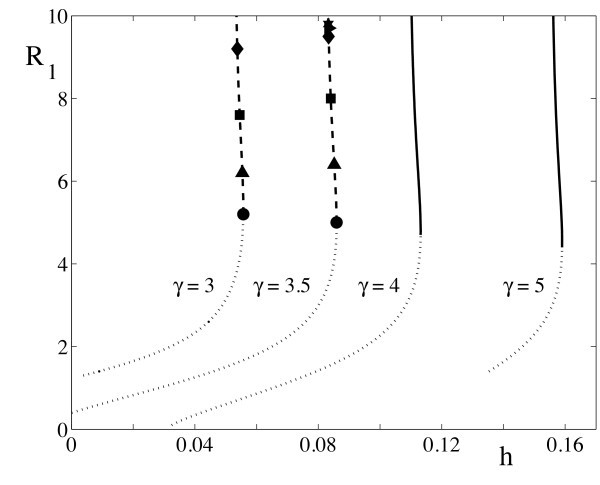
Existence and stability of ring solutions with inner radius $R_{1}$, with the indicated *γ* and β=0.5 in (20). The lower branch is always unstable (*dotted lines*). On the *upper branch* the stable ring (*solid lines*) can lose stability with dominant mode=2 (*solid circles*), 3 (*triangles*), 4 (*squares*), etc. for decreasing *h*.

**Fig. 6:**
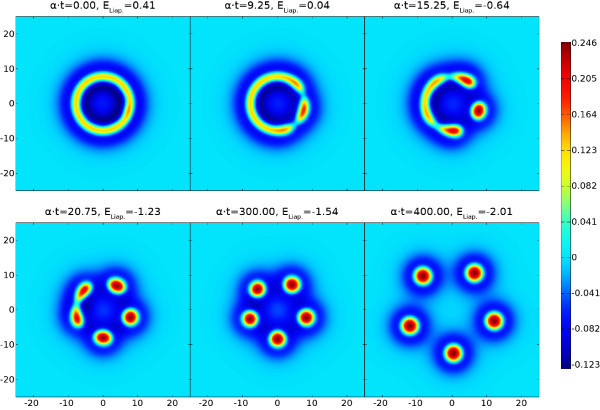
Direct numerical simulation of *u* for a ring solution ($R_{1}=7$, $R_{2}=8.629$) perturbed with a linear combination of modes 0,1,2,…,8. For the given parameters β=0.5, γ=3 and Heaviside threshold h=0.0549, mode m=5 is most unstable, cf. Figure 5. See also the video in Additional file 4.

At a bifurcation point defined by Reλm=0 we expect a ring to split into *m* spots. In Figure 5 we plot solution branches for ring solutions as a function of *h* for the Mexican-hat model defined by (20), and flag the types of instability that can occur. Of the two solution branches the lower one is unstable with respect to radial perturbations, whereas the upper branch is subject to azimuthal destabilizations. In Figure 6 we show a two-dimensional plot of an unstable ring solution, and the emergent structure of five bumps seen beyond instability, consistent with the predictions of our linear stability analysis. 

### 5.2 Fronts

Calculating stationary planar fronts is straightforward, since the normal vector n(s) is orthogonal to the displacement vector $r(s)-r(s^{\prime })$ and the line integral on the right hand side of (33) is zero. Hence we have the existence condition $h=\sum_{i}A_{i}\pi /\alpha_{i}^{2}$ and we note that *h* lies exactly halfway between the two possible steady states of *u*. To investigate the properties of a planar traveling front of speed *c* it is informative to treat the simple case $w(x)=K_{0}(x)/(2\pi )$. We then have that $u_{t}=-h+1/2$ and

(50) $$|z|=\int_{0}^{\infty }dse^{-s}\int_{-\infty }^{\infty }dy\frac{1}{2\pi }K_{0}\left(\sqrt{y^{2}+{\left(cs\right)}^{2}}\right)=\frac{1}{2}\frac{1}{1+c},\;c>0.$$

Hence using (22), the normal velocity is given by

(51) $$c=\frac{1-2h}{2h}.$$

To determine stability we consider a front along y=0 and write the perturbed front as $\hat{y}=\hat{y}(x,t)$.

For simplicity we shall focus on a stationary front with c=0. In this case we may construct δu(t) as

(52) $$\delta u(t)=\int_{0}^{\infty }dse^{-s}\int_{-\infty }^{\infty }dx^{\prime }\left[\hat{y}(x,t)-\hat{y}\left(x^{\prime }-x,t-s\right)\right]w\left(x^{\prime }\right).$$

The equation δu(t)=0 (for all *x*) has solutions of the form $\hat{y}=\epsilon \cos(kx)e^{\lambda t}$, where

(53) $$\lambda =-1+\frac{\hat{w}(k)}{\hat{w}(0)},\;\hat{w}(k)=\int_{-\infty }^{\infty }dxw(x)\cos(kx).$$

For a modified Bessel function one has

(54) $$\int_{-\infty }^{\infty }dxK_{0}(\alpha x)\cos(kx)=\frac{1}{\sqrt{\alpha^{2}+k^{2}}}.$$

**Fig. 7:**
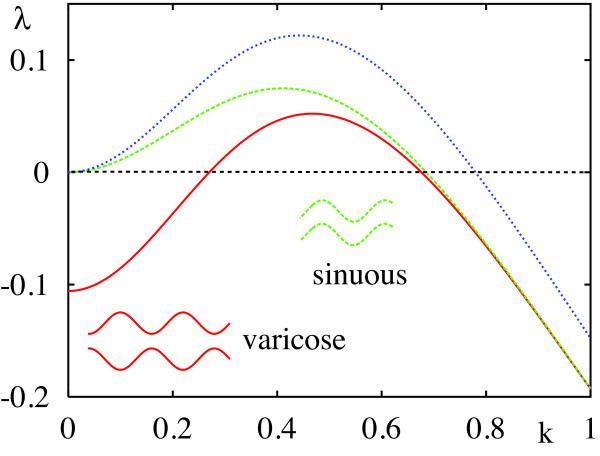
Spectra for the Mexican-hat function (20) with β=1/2 and γ=4: for a stationary front (*blue line*, $2h=1-1/(\gamma \beta^{2})$), and varicose (*green line*) and sinuous (*red line*) stripes of width D=7, respectively.

Hence for the simple example above, the stationary planar front is stable due to $\hat{w}(k)\leq \hat{w}(0)$. However, for a Mexican-hat function it is possible that $\hat{w}(k)>\hat{w}(0)$ for some band of wave numbers, and we would expect instabilities in this case. Figure 7 shows λ=λ(k) for a stationary front with the Mexican-hat function (20), from which the critical band of wave numbers can easily be read off. 

### 5.3 Stripes

A stripe may be considered as the active area in between two interacting stationary fronts. For two interfaces that define a stripe to be along $y=y_{1}$ and $y=y_{2}$, then

(55) $$u(x,y)=\int_{-\infty }^{\infty }dx^{\prime }\int_{y_{1}-y}^{y_{2}-y}dy^{\prime }w\left(\sqrt{{\left(x^{\prime }\right)}^{2}+{\left(y^{\prime }\right)}^{2}}\right).$$

For a stripe of constant width *D*, such that $y_{2}-y_{1}=D$ for all *x*, the existence condition $u(x,y_{1})=h=u(x,y_{1}+D)$ takes the simple form

(56) $$h=\int_{-\infty }^{\infty }dx\int_{0}^{D}dyw\left(\sqrt{x^{2}+y^{2}}\right)=\sum_{i}\frac{A_{i}\pi }{\alpha_{i}^{2}}\left[1-e^{-\alpha_{i}D}\right].$$

**Fig. 8:**
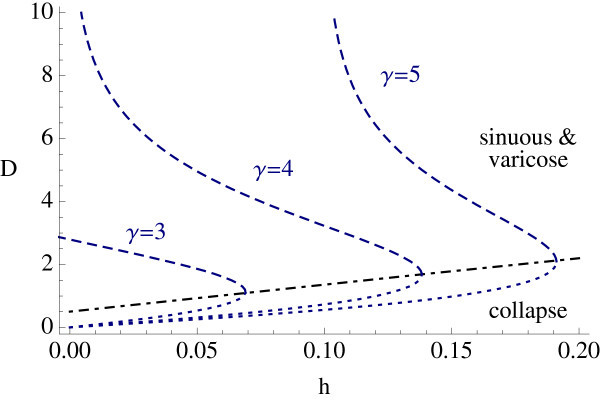
Stripe widths *D* for different values of *γ* with a Mexican hat interaction given by (20) and β=0.5.

An example of D=D(h) is shown in Figure 8 for a Mexican-hat function. 

To determine stability we consider perturbations on each of the two stripe boundaries and construct $\delta u_{i}$ on each as

(57) $$\delta u_{i}(t)=\int_{0}^{\infty }dse^{-s}\int_{-\infty }^{\infty }dx^{\prime }\left\{\int_{{\hat{y}}_{2}(x^{\prime }-x,t-s)-{\hat{y}}_{i}(x,t)}^{{\hat{y}}_{1}(x^{\prime }-x,t-s)-{\hat{y}}_{i}(x,t)}-\int_{0}^{D}\right\}\;dy^{\prime }w\left(\sqrt{{\left(x^{\prime }\right)}^{2}+{\left(y^{\prime }\right)}^{2}}\right),$$

for i=1,2. When considering small perturbations there is some ambiguity in expanding (57) depending on the relative size of the perturbations on each boundary. However, this at most amounts to a sign difference, which means that we may expand (57) to obtain

(58) $$\begin{array}{rl} \delta u_{i}(t) & =\int_{0}^{\infty }dse^{-s}\int_{-\infty }^{\infty }dx^{\prime }w\left(\sqrt{{\left(x^{\prime }\right)}^{2}+D^{2}}\right)\left[{\hat{y}}_{2}\left(x^{\prime }-x,t-s\right)-{\hat{y}}_{i}(x,t)\right]\\ & \;\pm \int_{0}^{\infty }dse^{-s}\int_{-\infty }^{\infty }dx^{\prime }w\left(x^{\prime }\right)\left[{\hat{y}}_{1}\left(x^{\prime }-x,t-s\right)-{\hat{y}}_{i}(x,t)\right]. \end{array}$$

The equations $\delta u_{i}=0$ admit solutions of the form ${\hat{y}}_{i}=y_{i}+\epsilon_{i}\cos(kx)e^{\lambda t}$. For equal amplitude perturbations, $|\epsilon_{1}|=|\epsilon_{2}|=\epsilon$, there are two branches of eigenvalues given by $\lambda =\lambda_{\pm }$, where

(59) $$\lambda_{\pm }=-1+\frac{F_{\pm }(k,D)}{F_{+}(0,D)}.$$

Here $F_{\pm }(k,D)=\hat{w}(k,0)\mp \hat{w}(k,D)$ and

(60) $$\hat{w}(k,D)=\int_{-\infty }^{\infty }dxw\left(\sqrt{x^{2}+D^{2}}\right)\cos(kx)=\sum_{i}A_{i}\frac{e^{-D\sqrt{\alpha_{i}^{2}+k^{2}}}}{\sqrt{\alpha_{i}^{2}+k^{2}}}.$$

**Fig. 9:**
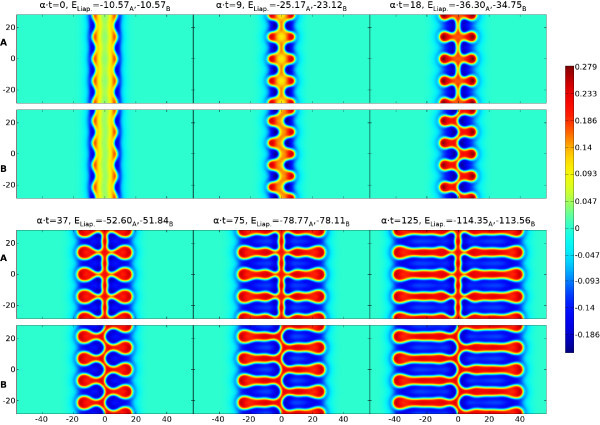
Direct numerical simulation of *u* for varicose (rows **A**) and sinuous (rows **B**) instabilities ±cos(ky) with k=0.44272 of a stripe of width D=6.08 for parameters β=0.5, γ=4.0 and Heaviside threshold h=0.03. See also the video in Additional file 5.

The branch with $\lambda =\lambda_{+}$ corresponds to sinuous perturbations with $\left(\epsilon_{1},\epsilon_{2})=\epsilon (1,1\right)$ and the branch with $\lambda =\lambda_{-}$ corresponds to varicose perturbations with $\left(\epsilon_{1},\epsilon_{2})=\epsilon (1,-1\right)$. Since $\lambda_{+}>\lambda_{-}$ then sinuous instabilities dominate over varicose. Note that as D→∞ we recover the existence and stability results for a stationary front as expected. Examples of sinuous and varicose instabilities (as predicted from our analysis) are shown in Figure 9. 

## 6 Neural field models with linear adaptation

 In real cortical tissues there are an abundance of metabolic processes whose combined effect is to modulate neuronal response. It is convenient to think of these processes in terms of local feedback mechanisms that modulate synaptic currents. For example, it is known that a model of synaptic depression can destabilize a spot in favor of a traveling pulse [25]. Here we consider a simple linear model of adaptation that is known to lead to instabilities of localized structures [26]. In this case the original neural field model is modified according to

(61) $$\frac{1}{\alpha }u_{t}=-u+\psi -ga,\;a_{t}=u-a,$$

with g>0. Here *ψ* is the second term on the right hand side of (3) and (1) in one and two dimensions, respectively. The linearity of the equations of motion means that we may obtain the trajectory for (u,a) in closed form as

(62) $$u(\cdot ,t)=\int_{-\infty }^{t}ds\eta (t-s)\psi (\cdot ,s),\;a(\cdot ,t)=\int_{-\infty }^{t}dse^{-(t-s)}u(\cdot ,s),$$

where

(63) $$\eta (t)=\frac{\alpha }{\lambda_{-}-\lambda_{+}}\left\{(1-\lambda_{+})e^{-\lambda_{+}t}-(1-\lambda_{-})e^{-\lambda_{-}t}\right\}.$$

Here

(64) $$\lambda_{\pm }=\frac{1+\alpha \pm \sqrt{{\left(1+\alpha \right)}^{2}-4\alpha (1+g)}}{2}.$$

As an example let us compute the speed (c>0) and stability of a front in the one-dimensional model discussed in Section 2 with the inclusion of a linear adaptation current. In this case we have that

(65) $$\begin{array}{r} u_{t}{|}_{x=x_{0}(t)}=ch/\sigma , \end{array}$$

(66) $$\begin{array}{rl} u_{x}{|}_{x=x_{0}(t)} & =-\frac{1}{c}\int_{0}^{\infty }ds\eta (s/c)w(s)\\ & =-\frac{\alpha }{c(\lambda_{-}-\lambda_{+})}\left[(1-\lambda_{+})\tilde{w}(\lambda_{+}/c)-(1-\lambda_{-})\tilde{w}(\lambda_{-}/c)\right]. \end{array}$$

Note that to calculate $u_{t}$ we have used the result that $h=u(ct,t)=\int_{0}^{\infty }ds\eta (s)\int_{cs}^{\infty }dyw(y)$. Hence, from (5), the speed is determined implicitly by

(67) $$h=\frac{\alpha }{2}\frac{c/\sigma +1}{\left(c/\sigma +\lambda_{+})(c/\sigma +\lambda_{-}\right)},$$

which may be rearranged to give

(68) $$\frac{c}{\sigma }=-\frac{1}{2}\left[1+\alpha -\frac{\alpha }{2h}\pm \sqrt{{\left(1+\alpha -\frac{\alpha }{2h}\right)}^{2}-4\alpha \left(1+g-\frac{1}{2h}\right)}\right].$$

The eigenvalue equation for stability can also be calculated, generalizing the analysis of Section 2, as E(λ)=1−H(λ)/H(0), where

(69) $$H(\lambda )=\frac{\alpha }{\lambda_{-}-\lambda_{+}}\left\{(1-\lambda_{+})\tilde{w}\left((\lambda +\lambda_{+})/c\right)-(1-\lambda_{-})\tilde{w}\left((\lambda +\lambda_{-})/c\right)\right\}.$$

On the branch with c=0 where 2h(1+g)=1, defining a stationary front, we find that

(70) $$E(\lambda )=\lambda \frac{\left(\lambda +\lambda_{+}+\lambda_{-}-\lambda_{+}\lambda_{-}\right)}{\left(\lambda +\lambda_{+})(\lambda +\lambda_{-}\right)},$$

which has zeros when λ=0 and $\lambda =k_{+}k_{-}-(k_{+}+k_{-})=\alpha g-1$. Hence, the stationary front changes from stable to unstable as *α* is increased through $\alpha_{c}=1/g$.

In two-dimensions it is straight forward to construct a stationary spot of radius *R*. This radius is determined by (38) under the replacement h→h(1+g), so that

(71) $$h(1+g)=2\pi R\sum_{i=1}^{N}A_{i}K_{0}(\alpha_{i}R)I_{1}(\alpha_{i}R)/\alpha_{i}\equiv F(R).$$

Here *ψ* may be constructed explicitly off the boundary, and is given by Equation (41), so that u(r)=ψ(r)/(1+g). A saddle-node bifurcation of stationary spots occurs at $R=R_{c}$ where $F^{\prime }(R_{c})=0$. Hence, in the (h,g) plane stationary solutions only exist for $h<F(R_{c})/(1+g)$. Under variation in *α* we expect the emergence of a drifting spot. Beyond a drift instability, we expect to be able to find traveling spots that move in some direction **c** with constant speed c=|c|. These can be constructed as stationary solutions in a co-moving frame ξ=x+ct, and satisfy

(72) $$\frac{1}{\alpha }c\cdot \nabla_{\xi }u=-u+\psi -ga,\;c\cdot \nabla_{\xi }a=u-a.$$

We may write the velocity in terms of local co-ordinates on the moving interface as $c=c_{n}n+c_{t}t$, where $c_{n}(s)=c\cdot n(s)$ is the normal velocity and $c_{t}(s)=c\cdot t(s)$ the tangential velocity at a point on the interface. Taking the cross product of **c** and **t** (and using n×t=1) shows that $c_{n}=c\times t$. Hence, the condition for stationary propagation, with $c=\dot{r}$, is

(73) $$n(s)\cdot \dot{r}(s)=c\times \frac{dr(s)}{ds}/|\frac{dr(s)}{ds}|,\;u(\xi ){|}_{\xi =r}=h.$$

In general this is a hard equation to solve in closed form. However, to obtain an estimate of the speed and shape of a spot beyond a point of instability it is enough to consider a weak distortion of a traveling circular wave [27]. Choosing c=c(1,0) and writing $\xi =(\xi_{1},\xi_{2})$, and assuming that *ψ* is rotationally symmetric means that we may construct a solution in the form

(74) $$u(\xi_{1},\xi_{2})=\frac{1}{c}\int_{-\infty }^{\xi_{1}}dy\eta \left((\xi_{1}-y)/c\right)\psi \left(\sqrt{y^{2}+\xi_{2}^{2}}\right).$$

We note that the threshold condition u=h for a circular spot ($\xi_{1}^{2}+\xi_{2}^{2}=R^{2}$) can only strictly be met for the case c=0, since the right hand side of (74) depends on the plane polar angle through $\xi_{1}=R\cos\theta$. For this case we may construct the equation δu(t)=0 to determine the eigenvalues $\lambda_{m}$ that occur in perturbations of the form $\delta R(\theta ,t)=\epsilon e^{\lambda_{m}t}\cos(m\theta )$ as solutions to $E_{m}(\lambda )=0$, where

(75) $$E_{m}(\lambda )=\frac{1}{\tilde{\eta }(\lambda )}-(1+g)W_{m},$$

where $W_{m}$ is given by (44) and the Laplace transform of *η* is easily calculated as

(76) $$\tilde{\eta }(\lambda )=\frac{\alpha (1+\lambda )}{\left(\lambda +\lambda_{+})(\lambda +\lambda_{-}\right)}.$$

**Fig. 10:**
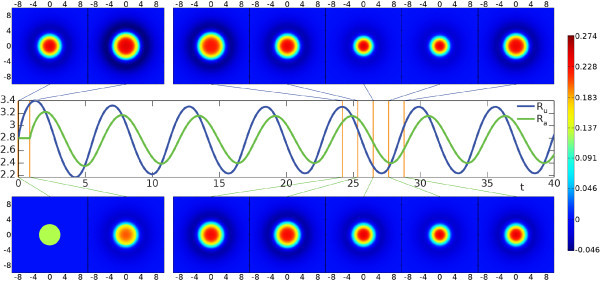
Breathing instability from direct numerical simulation with parameters α=5, β=0.5, γ=4, coupling strength g=0.5 and Heaviside threshold h=0.12/(1+g). Initial data was constructed from a stationary spot solution by modifying the adaptation variable to a top-hat shape. *Top* and *bottom rows*: snapshots of activation *u* and adaptation *a*, respectively, at times indicated by orange lines in the middle row. *Middle row*: above threshold radii as function of time. See also the video in Additional file 6.

The eigenvalues for m=1 are determined by $\tilde{\eta }(\lambda )=1/(1+g)$, which has two solutions: λ=0 and λ=αg−1. Hence this mode becomes unstable as *g* increase through 1/α. It is also possible that a breathing instability may arise for the mode with m=0. Note that another way to generate breathing solutions is to include localized inputs [3,4], breaking the homogeneous structure of the network. Substitution of λ=iω into (75) gives the condition for this instability as:

(77) $$W_{0}=\frac{1+\alpha }{\alpha (1+g)},\;g\geq \frac{1}{\alpha },$$

with emergent frequency $\omega =\sqrt{\alpha g-1}$. For m≥2 splitting instabilities can be determined by setting λ=0 in (75) to give the conditions $W_{m}=1$. An example of a breather arising as an instability of a spot is shown in Figure 10 (and numerical simulations confirm the predicted value of the emergent frequency around the bifurcation point). 

Anticipating a small *c* discussion we Laplace transform (74) in the $\xi_{1}$ variable to obtain

(78) $$\tilde{u}(\lambda ,\xi_{2})=\frac{1}{1+g}\left(1+\frac{1}{\alpha (1+g)}\left[(\alpha g-1)c\lambda -\alpha {\left(c\lambda \right)}^{2}+{\left(c\lambda \right)}^{3}+\cdots \right]\right)\tilde{\psi }(\lambda ,\xi_{2}),$$

which we then inverse transform to obtain

(79) $$\begin{array}{rl} u(\xi_{1},\xi_{2}) & =\frac{1}{1+g}\{1+\frac{1}{\alpha (1+g)}[c(\alpha g-1)\frac{\partial }{\partial \xi_{1}}\\ & \;-\alpha c^{2}\frac{\partial^{2}}{\partial \xi_{1}^{2}}+c^{3}\frac{\partial^{3}}{\partial \xi_{1}^{3}}+\cdots ]\}\psi \left(|\xi |\right). \end{array}$$

At the point where g=1/α, the shape of the spot deviates from circular with an amplitude that depends on quadratic and higher powers of *c*. Thus not only is there a breathing bifurcation at g=1/α, but also a drifting instability to a traveling spot whose shape, determined from (79) by u(r)=h, can be written in the form r(θ)=R(θ)(cosθ,sinθ) with

(80) $$R(\theta )=R+\sum_{m\geq 2}c^{m}a_{m}\cos m\theta .$$

Here *R* is determined by (71). A further weakly nonlinear analysis to understand the competition between drifting and breathing at g=1/α is beyond the scope of this article.

**Fig. 11:**
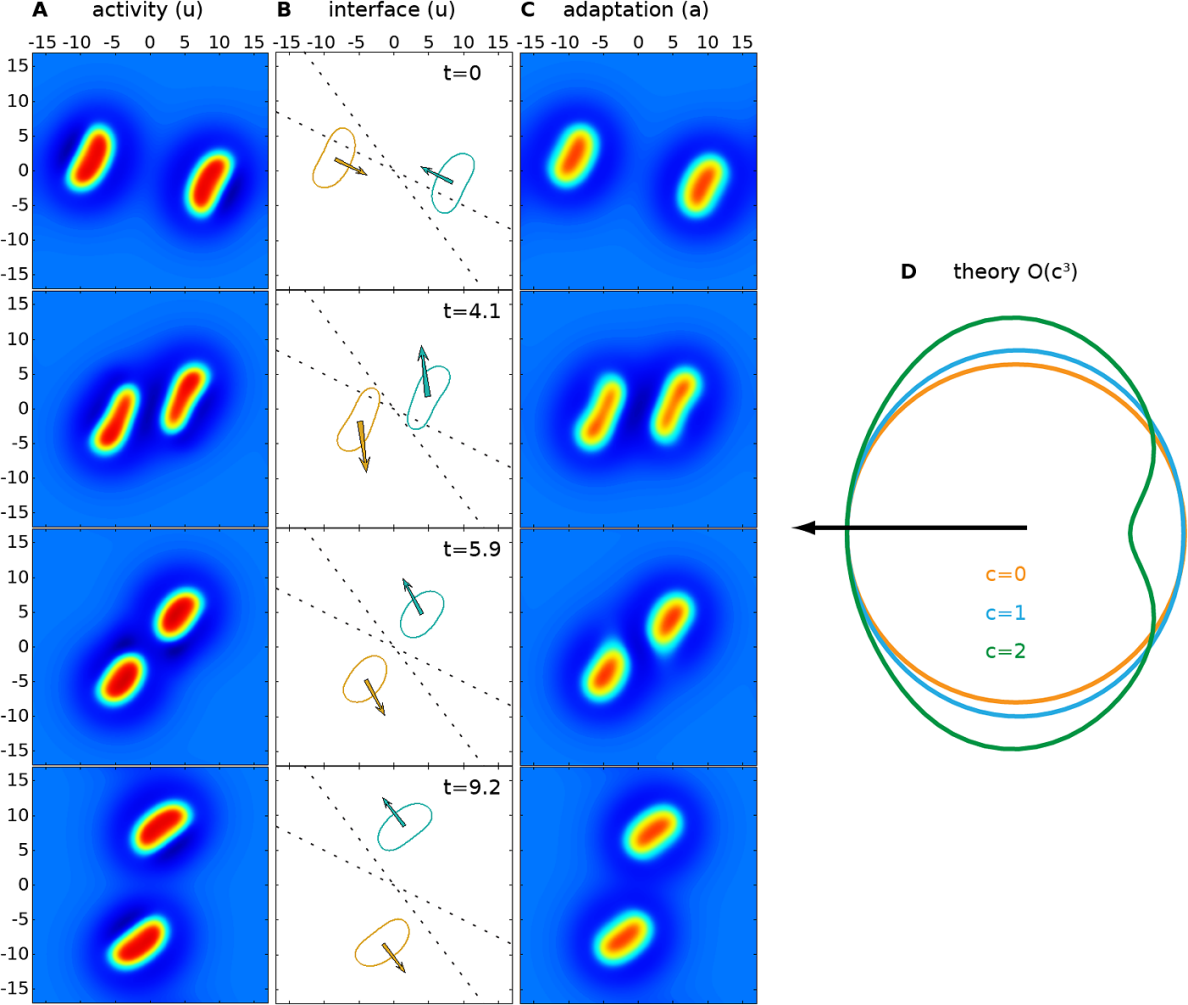
Collision of two pulses from direct numerical simulation with parameters as in Figure 10. Columns **A** and **C** show *u* and *a*, respectively. Column **B** shows the u=h interfaces, velocities (arrows to scale but enlarged by a factor of four), and *dotted centerlines* as visual guides. See also the video in Additional file 7. Column **D** predicts to $O(c^{3})$ the shape of a traveling pulse for different *c*.

For g>1/α and dropping terms of $O(c^{2})$ in (79) we see that there are solutions to u(r)=h of the form $R(\theta )=R+a_{1}c\cos\theta$, where $a_{1}=(1-\alpha g)/(\alpha {\left(1+g\right)}^{2})<0$. The amplitudes of higher order modes may be constructed in a similar fashion, i.e., by balancing terms at each order in *c* in u(r)=h using (79) and (80). However, it is not our intention to pursue these lengthy calculations here. Rather to give a feel of the shape of a traveling spot we plot the level set where $u(\xi_{1},\xi_{2})=h$ using (79) in Figure 11D including terms up to $c^{3}$. This nicely illustrates that spots contract in the direction of propagation and widen in the orthogonal direction, and provides a theoretical explanation for the shape of traveling spots recently reported in [28]. With the aid of direct numerical simulations we have also explored the scattering properties of traveling spots. In common with previous numerical studies of planar neural fields with some form of adaptation, we find that such structures can behave as quasi-particles in the sense that they can scatter like dissipative solitons [29]. An example of such scattering is shown in Figure 11. Here we see a repulsive interaction which repels the spots away from each other if they approach too closely. 

## 7 Discussion

 In this article we have formulated an interface dynamics for planar neural fields with a Heaviside firing rate. This has allowed us to (i) develop an economical computational framework for the evolution of spatiotemporal patterns, and (ii) perform linear stability analyses of localized structures. For simplicity we have focused on single population models. However, the extension to population models that treat the dynamics of both excitatory and inhibitory populations is straightforward. Perhaps a more interesting extension is to consider neural field models that incorporate feature selectivity such as that observed in visual cortex for orientation [30], spatial frequency [31] and texture [32]. Denoting this feature label by *χ* then all of these models are expressed in terms of some non-local integro-differential equation for u(r,χ,t). We note that the notion of an interface is still well defined and that the level set condition u(r,χ,t)=h gives a constraint between local geometrical data and features. As an alternative to simulating the neural field models an interface approach (incorporating feature space) may be more useful for understanding how local data can be integrated into global geometrical structures, as advocated in the neurogeometry framework of Petitot [33] (say for understanding models of contour completion in models of primary visual cortex where the feature space is orientation). The extension of this work to treat sigmoidal firing rates remains an open challenge. However, recent techniques for dealing with a certain class of firing rate functions in one spatial dimension, which includes smooth firing rate functions connecting zero to one, are likely to be useful in this regard [34]. We have included an adaptive current in the standard Amari model here, but it would be informative to develop interface treatments for other forms of modulation, e.g., arising from threshold accommodation [35] or synaptic depression [5], as well as the inclusion of axonal delays [36]. These models can readily support spiral wave activity, and it would be interesting to see if an interface description, possibly adapting techniques by Hagberg and Meron [37], could shed light on their properties. Another possible extension of the work in this article, motivated by our numerical results for scattering spots, is to develop an interface theory of quasi-particle interactions along the lines for reaction-diffusion models described in [38,39], using ideas developed by Bressloff [40] and Venkov [41] for weakly interacting systems in one spatial dimension. All of the above are topics of ongoing research and will be reported upon elsewhere. 

## Appendix: Numerical schemes

### A.1 Fourier technique for neural field evolution

Because of its non-local character, the model described by (1), or its extension (61), is challenging to solve with conventional numerical methods. However, exploiting the convolution structure of (1) allows one to write the Fourier transform of $\int_{R^{2}}dx^{\prime }w(|x-x^{\prime }|)f(x^{\prime },t)$ as a product. Here f(x,t)=H(u(x,t)−h) and can be taken either as a Heaviside or a more general sigmoidal form. Introducing a spectral wave-vector **k** then this product is simply w(|k|)f(k), where functions with arguments **k** denote two-dimensional spatial Fourier transforms. We may evaluate w(|k|)f(k) directly, at every time step, using fast Fourier transforms (FFTs). Note that w(|k|) can be pre-computed, by FFT or here even analytically, so that the procedure iterated over time amounts to computing f(k) by FFT, followed by a (complex) multiplication with w(|k|), and finally an inverse FFT to obtain the result of the integral. We wish to employ a parallel compute cluster for rapid computation over large grids, and hence use the free software package FFTW 3.3 [42], which includes a parallel MPI-C version. Note that the use of Fourier methods implies that the discretization grid has periodic boundaries, or in other words, the solution is effectively computed on a torus. We use a grid spacing of about 0.03 or better in our computations here. 

 In order to compute the time evolution, we use DOPRI5 [43], a well-known implementation of an explicit Dormand-Prince (Runge-Kutta) method of order 5(4) with step size control and dense output of the order 4. A version in C due to J. Colinge is available on the web thanks to E. Hairer. However, in our case we perform parallel computations, so we have adapted this code accordingly using MPI-C. In particular, we now consider the maximum error across all compute nodes and all variables, rather than the mean error over local variables, and communicate the resulting time step adaptation over the cluster to achieve a unified evolution of the entire distributed grid. Numerical tolerances are set to $10^{-7}(|y_{i}|+1)$ where $y_{i}$ represents all variables, i.e., *u* and potentially *a* at all grid points.

This numerical method is robust against effects of the underlying grid. This is due to the employed Fourier method, which performs the spatial convolution as a multiplication in Fourier space. The discrete Fourier transform used to transfer this calculation to Fourier space calculates a trigonometric interpolation polynomial, and the influence of the grid is effectively smoothed by implicit interpolation.

Computing an evolution as shown in Additional File 1 takes several hours on the 32 to 64 Infiniband-connected compute nodes we have typically employed, and yields many gigabytes of data. We note that computation with a sigmoidal firing rate instead of the Heaviside one is over an order of magnitude faster, reflecting the numerical difficulty of dealing with sharp edges.

### A.2 Interface dynamics

Equations (22) and (28) can be used to develop a numerical scheme. The contour ∂B is discretized into a set of points, and the normal vectors and the displacement vectors are found by computing the orientation and distance between points. Hence the computation of the contour integrals in (28) is straight-forward and yields the normal velocity, cf. (22), which is used to displace the points of the contour in the normal direction at every time step. We employed a simple Euler method to calculate the dynamics of the contour. As the contour grows/shrinks, additional points have to be created/eliminated along the contour.

This method does not provide any means to deal with the splitting or emergence of contours. It is faster than the Fourier technique (see Section A.1 in the Appendix) for small contours, yet the time to compute the normal velocity is proportional to $N^{2}$ (*N* being the number of points discretizing the contour), as opposed to $M\sqrt{M}$ for the Fourier technique (where *M* is the number of grid points). Hence it becomes slower for larger contours due to the absence of suitable spectral methods to compute the line integrals. The main advantage of this method is the fact that no underlying grid has to be deployed across the specified domain.

## Electronic Supplementary Material

## Competing interests

The authors declare that they have no competing interests.

## Author’s contributions

SC, HS and IB contributed equally. All authors read and approved the final manuscript.

## Supplementary Material

Additional file 1 A movie of an evolving labyrinthine structure. Emergence of a labyrinthine structure in *u* as shown in snapshots in Figure 1. Panels A and B in the animation correspond to rows A and B of that figure, and displayed content, parameters and initial condition are discussed in its caption. The 120×120 domain was discretized by a 4,096×4,096 grid. Note that the Fourier technique used, see Section A.1 in the Appendix, implies periodicity and turns the domain effectively into a torus. (AVI 16.0 MB) Click here for file

Additional file 2 A movie showing the similarity between Heaviside and steep sigmoidal models. In panel A the values of *u* from three direct numerical simulations are overlaid by assigning each one a part of the red-green-blue color space. The Heaviside model, cf. Figure 1 and the video in Additional file 1, determines red intensity, whereas calculations with a sigmoidal firing rate 1/{1+exp[−(u−h)/σ]}, cf. Figure 3, determine intensities in green for σ=0.01 and blue for σ=0.02. Where all three models predict the *same* value for *u*, a gray color results as shown by the colorbar. Where the models predict different values, colored patches show up. In panel B the same data is displayed, but this time only the u=h interface is plotted with the same color assignment. From about time α⋅t=550 onward the structure starts to interfere with itself across the grid edges. (AVI 18.0 MB) Click here for file

Additional file 3 A movie showing pattern evolution for a shallow sigmoidal model. The same model as in Figure 1 but with a sigmoidal firing rate 1/{1+exp[−(u−h)/σ]} with σ=0.03. A comparison with the Heaviside model, Figure 1 and the video in Additional file 1, as well as the sigmoid model with smaller *σ*, Figure 3 and the video in Additional file 1, shows that broadening the sigmoid eventually leads to significant deviations from the Heaviside prediction. This illustrates the practical limits of the interface method proposed in this article. (AVI 17.0 MB) Click here for file

Additional file 4 A movie of a fivefold spot pattern arising from a ring instability. Decay of a perturbed ring solution in *u* into five spots as shown in snapshots in Figure 6. Displayed content, parameters and initial condition are discussed in that figure’s caption. The 50×50 domain was discretized by a 2,048×2,048 grid. Note that the simulation time (displayed on top of the *u* plot) is nonlinearly related to the actual play time of the animation, in order to capture both the rapid structural change from ring to spots at the beginning and the slow drifting apart of the spots that follows. (AVI 12.0 MB) Click here for file

Additional file 5 A movie showing fingers of instability. Varicose and sinuous instabilities of a stripe as shown in snapshots in Figure 9. Panels A and B in the animation correspond to rows A and B of that figure, and displayed content, parameters and initial condition are discussed in its caption. For the present set of parameters varicose instabilities occur for 0.27<k<0.69, whereas sinuous instabilities occur for 0<k<0.69. The domain size is set to 8π/k in the ordinate to guarantee that perturbations ±cos(ky) are periodic. The domain size in the abscissa is 16π/k and the domain was discretized by a 4,096×2,048 grid. (AVI 10.0 MB) Click here for file

Additional file 6 A movie showing a breather. Breathing spot as shown in snapshots in Figure 10. Panels A and B in the animation correspond to the top and bottom row of that figure, and displayed content and parameters are discussed in its caption. Both activation *u* and adaptation *a* start with radius R=2.8 above threshold, the former with a spot solution and the latter with a disc of constant value 0.25*g*. The spot oscillates with angular frequency ω=1.1, close to the theoretical prediction from linear stability analysis ($\omega =\sqrt{\alpha g-1}≃1.2$, with increasing agreement as one approaches the bifurcation point αg=1 from above). The actual domain was 34×34, of which only part is shown, and was discretized by a 1,024×1,024 grid. (AVI 7.7 MB) Click here for file

Additional file 7 A movie showing spot scattering. Collision of two travelling spots as shown in snapshots in Figure 11. Panels A-C in the animation correspond to rows A-C of that figure, and displayed content and parameters are discussed in its caption. Note that time t=0 in the figure corresponds to t=36.95 in the animation. To initially create the traveling spots, two spot solutions in *u* with radius 2.8 are used, with two co-located discs in *a* of the same radius. The discs have a linear gradient along the abscissa from zero to 0.5*g*, while having uniform values along the ordinate. The mean position of the u≥h regions of the pulses is kept track of during time evolution and used to estimate the current velocities. The 34×34 domain was discretized by a 1,024×1,024 grid. (AVI 17.0 MB) Click here for file
